# A Quality Improvement Project to Evaluate Satisfaction With Alternatives to Face to Face Consultation in a Learning Disability Service

**DOI:** 10.1192/bjo.2022.291

**Published:** 2022-06-20

**Authors:** Eslam Elmasry, Catherine Bright

**Affiliations:** Wales Deanery, Newport, United Kingdom

## Abstract

**Aims:**

COVID-19 pandemic has had a great impact on all groups in society. People with intellectual disability (ID)/learning disability (LD) are especially vulnerable. As a result, restrictions were put in place to protect this group, including limiting face to face visits/consultations. Restrictions on usual activities of people with learning disability are likely to induce stress leading to an escalation in challenging behaviors. Regular assessments and follow-ups are essential to evaluate the patients and provide the best care, so virtual consultations (via telephone or video call) were identified as a potential alternative to face-to-face consultations

Aim: Evaluation of the service provided during the COVID-19 pandemic including virtual clinics.

**Methods:**

A questionnaire was designed to evaluate the patients and their careers’ satisfaction with the virtual clinics, seeking their feedback about positives and limitations of the service and exploring their preferences for future clinical contact. Data were collected during May 2021. Different professions including (psychiatrists, psychologists, nurses, occupational therapists and speech and language therapists) in community services for adults with learning disabilities in Aneurin Bevan University Health Board have participated in the survey. The questionnaires were filled by service users, their carers or by the service provider.

Numbers of DNA (Did not attend) across the whole service during May 2021 were compared to DNAs in May 2018, 2019 and 2020.

**Results:**

140 surveys were completed. Patients and their carers were happy with many aspects of the service provided through the pandemic. It was reported that virtual clinics are an efficient way to meet with professional carers and families where there are difficulties bringing patients to clinics, however home visits were preferred for assessing patients.

No noticeable change in DNA rates has been identified.

**Conclusion:**

Virtual clinics have been well tolerated by patients and their carers during the pandemic and have provided an extremely efficient tool to overcome the restrictions which were imposed.

Carers and patients expressed satisfaction with clinic appointments provided remotely.

